# Malignant melanoma after treatment for Merkel cell carcinoma^☆^^☆☆^

**DOI:** 10.1016/j.abd.2020.02.013

**Published:** 2020-07-15

**Authors:** Masato Ishikawa, Toshiyuki Yamamoto

**Affiliations:** Department of Dermatology, Fukushima Medical University, Fukushima, Japan

Dear Editor,

A 98-year-old male was referred complaining of a mass on his left cheek. He had never received immunosuppressive therapy. Physical examination showed a solid, dome-shaped, reddish tumor, 35 mm in diameter, on his left cheek. Microscopic examination of a biopsy specimen revealed an infiltrative tumor in the dermis. The tumor was composed of sheets and trabeculae of atypical cells with scant cytoplasm, and round nuclei with stippled chromatin (Fig. 1). Immunohistochemistry results revealed that the tumor cells stained positive for cytokeratin 20 and neuron specific enolase. The patient underwent total resection of the tumor. However, because of the patient’s advanced age, neither the patient nor his family wished further treatment, including radiotherapy, choosing follow-up visits only. Two years later, he presented again with erosion of the right hallux. Physical examination showed widespread erosion of his right hallux, which in part had a reddish nodule. The nail had completely disappeared (Fig. 2). Histological examination revealed many atypical cells infiltrating irregularly from the epidermis to the dermis (Fig. 3). These atypical cells were immunoreactive for MART-1 and HMB-45. For the same reason as before, the patient and his family wanted only local symptomatic treatments at a nearby hospital.

**Figure 1:**
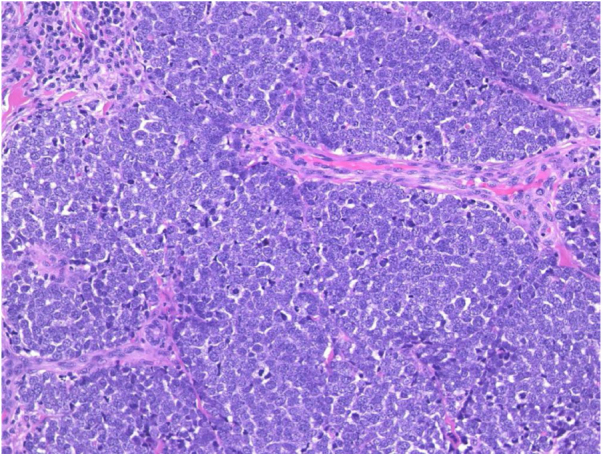
Microscopic examination of a biopsy specimen from a mass on patient’s left cheek. There was an infiltrative tumor in the dermis. The tumor was composed of sheets and trabeculae of atypical cells with scant cytoplasm, and round nuclei with stippled chromatin (Hematoxylin & eosin, ×200).

**Figure 2:**
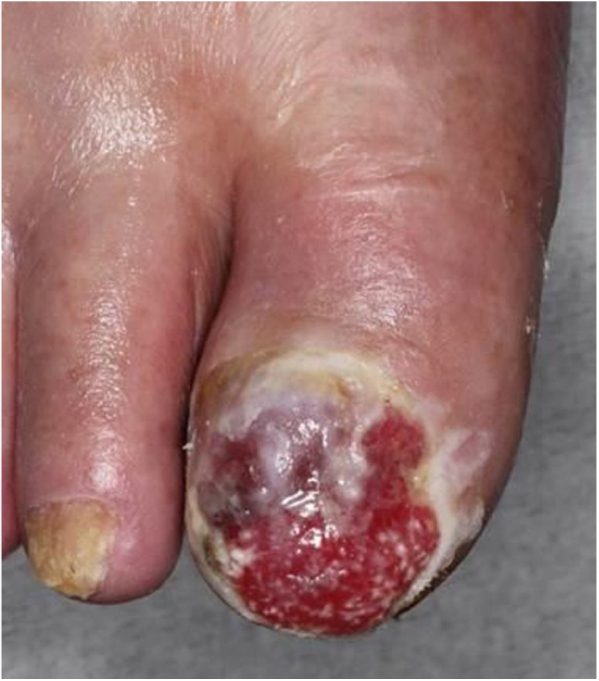
Physical examination revealed widespread erosion of the right hallux, which in part had a reddish nodule. The nail had completely disappeared.

**Figure 3:**
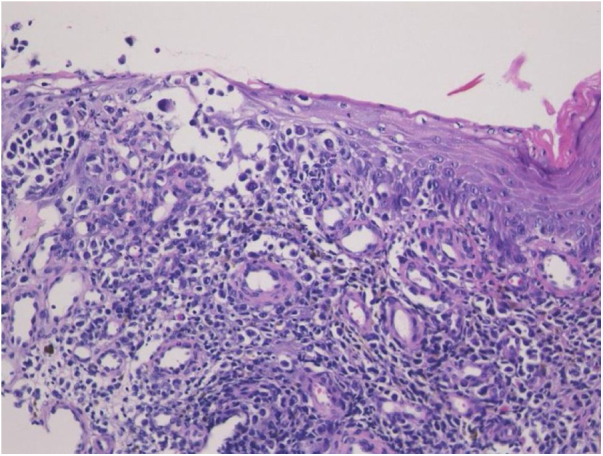
Histological examination showed many atypical cells infiltrating irregularly from the epidermis to the dermis (Hematoxylin & eosin, ×200).

Merkel cell carcinoma (MCC) is a rare cutaneous neuroendocrine cancer that is known to be a highly aggressive malignancy with frequent metastasis and a high mortality rate. A previous review article revealed increased risk of a second primary cancer after the diagnosis of MCC, and cutaneous malignant melanoma (MM) is one of the most common cancers after MCC.^1^ Miller and Rabkin reported that occurrences of MM and MCC were similarly increased with sun exposure and immunosuppression.^2^ Considering that ultra violet (UV) rays attenuate systemic immune responses *via* the induction of regulatory T-cells, systemic immunosuppression may be an essential factor in the development of both conditions in a patient.^3^ In the present case, MCC occurred on the left cheek, and MM occurred on the right hallux. In Japan, the majority of MM cases occur on the edges of limbs that are not directly affected by UV rays, and external irritation has been considered one of the risk factors.^4^ However, systemic immunosuppression associated with aging and/or UV irradiation may be a common cause of both conditions in the present case. According to previous articles, several cases in which MCC and MM developed in a patient have been reported from countries other than Japan; however, to the best of the authors’ knowledge, this is the first report of its type from Japan. Interestingly, there was a report of a case of MCC that developed in a patient during treatment with immune checkpoint inhibitors for MM.^2^, ^5^ Given the increase of immune checkpoint inhibitors used to treat MM, the number of cases in which both MCC and MM develop in a patient may increase in Japan, as well as around the world.

## Financial support

None declared.

## Authors' contributions

Masato Ishikawa: Drafting and editing of the manuscript; collection, analysis, and interpretation of data; intellectual participation in the propaedeutic and/or therapeutic conduct of the studied cases; critical review of the literature.

Toshiyuki Yamamoto: Approval of the final version of the manuscript; conception and planning of the study; critical review of the manuscript.

## Conflicts of interest

None declared.
